# Comparison of Brain Development in Sow-Reared and Artificially Reared Piglets

**DOI:** 10.3389/fped.2016.00095

**Published:** 2016-09-12

**Authors:** Reeba M. Jacob, Austin T. Mudd, Lindsey S. Alexander, Chron-Si Lai, Ryan N. Dilger

**Affiliations:** ^1^Piglet Nutrition and Cognition Laboratory, University of Illinois, Urbana, IL, USA; ^2^Division of Nutritional Sciences, University of Illinois, Urbana, IL, USA; ^3^Department of Animal Sciences, University of Illinois, Urbana, IL, USA; ^4^Neuroscience Program, University of Illinois, Urbana, IL, USA; ^5^Abbott Nutrition, Abbott Laboratories, Columbus, OH, USA

**Keywords:** animal model, diffusion tensor imaging, neurodevelopment, magnetic resonance imaging, piglet, sow-reared, voxel-based morphometry

## Abstract

**Introduction:**

Provision of adequate nutrients is critical for proper growth and development of the neonate, yet the impact of breastfeeding versus formula feeding on neural maturation has to be fully determined. Using the piglet as a model for the human infant, our objective was to compare neurodevelopment of piglets that were either sow-reared (SR) or artificially reared (AR) in an artificial setting.

**Methods:**

Over a 25-day feeding study, piglets (1.5 ± 0.2 kg initial bodyweight) were either SR (*n* = 10) with *ad libitum* intake or AR (*n* = 29) receiving an infant formula modified to mimic the nutritional profile and intake pattern of sow’s milk. At study conclusion, piglets were subjected to a standardized set of magnetic resonance imaging (MRI) procedures to quantify structure and composition of the brain.

**Results:**

Diffusion tensor imaging, an MRI sequence that characterizes brain microstructure, revealed that SR piglets had greater (*P* < 0.05) average white matter (WM) (generated from a piglet specific brain atlas) fractional anisotropy (FA), and lower (*P* < 0.05) mean and radial and axial diffusivity values compared with AR piglets, suggesting differences in WM organization. Voxel-based morphometric analysis, a measure of white and gray matter (GM) volumes concentrations, revealed differences (*P* < 0.05) in bilateral development of GM clusters in the cortical brain regions of the AR piglets compared with SR piglets. Region of interest analysis revealed larger (*P* < 0.05) whole brain volumes in SR animals compared with AR, and certain subcortical regions to be larger (*P* < 0.05) as a percentage of whole brain volume in AR piglets compared with SR animals. Quantification of brain metabolites using magnetic resonance spectroscopy revealed SR piglets had higher (*P* < 0.05) concentrations of myo-inositol, glycerophosphocholine + phosphocholine, and creatine + phosphocreatine compared with AR piglets. However, glutamate + glutamine levels were higher (*P* < 0.05) in AR piglets when compared with SR animals.

**Conclusion:**

Overall, increases in brain metabolite concentrations, coupled with greater FA values in WM tracts and volume differences in GM of specific brain regions, suggest differences in myelin development and cell proliferation in SR versus AR piglets.

## Introduction

Early-life nutrition, whether provided as human milk, infant formula, or a combination of the two, may influence structural brain development, as well as subsequent cognitive and behavioral development of the child. Epidemiological findings suggest variable impact between breastfeeding and formula feeding on motor development, problem solving skills, and social outcomes (1). An increasing number of neuroimaging and cognitive studies positively correlate the consumption of human milk and duration of breastfeeding with increased macrostructural maturation, improved white matter (WM) maturation, and enhanced performance on cognitive testing in adolescence (2, 3). Notably, the use of neuroimaging is relatively new in the field of infant nutrition research. A recent review suggests the positive impact of this technique in an already established field, but cautions that much of this early work is proof of principle to generate evidence for how diet can shape the neurodevelopmental trajectory (4). As such, characterization of breast-fed infant neurodevelopment is of primary interest, as these infants are often considered the normative standard for comparison in nutritional intervention studies.

Magnetic resonance imaging (MRI) techniques allow for a non-invasive means of assessing brain development throughout a period when brain growth is highly dynamic and susceptible to nutrient interventions. However, challenges in pediatric neuroimaging including subject anxiety and discomfort, inability to restrict movement, and the need for imaging during non-sedated sleep often make acquisition of useable data quite difficult (5). Thus, a number of studies capture these outcomes in older children (6–8), whereas characterization of the same phenomena in infants is just appearing in the literature (9, 10). Moreover, there is a dearth of neuroimaging studies that directly assess the difference between formula-fed and breast-fed infant brain development (2). Given the challenges of infant neuroimaging and the continued acceptance of animal models in nutritional neuroscience, there still exists a need to comprehensively characterize an appropriate animal model to study the impact of early life nutrition on the developing brain using neuroimaging procedures. Therefore, our research focuses on using the piglet as a model for infant nutrition and neurodevelopment.

Use of MRI techniques in the artificially reared (AR) piglet has previously established the piglet as a clinically relevant translational model for the human infant (11–17). We propose the AR piglet is similar to the formula-fed infant, while the sow-reared (SR) piglet as the equivalent of the breast-fed infant. While the AR piglet is well established in the field of nutritional neuroscience, characterization of SR piglet neurodevelopment remains to be elucidated, and comparison with AR piglet development is needed. Thus, analysis of SR piglet brain development will establish a normative standard, and therefore benefit current and future conspecific comparisons for structural, functional, and cognitive processes. As such, a primary objective of this study was to quantify the differences in brain macro- and microstructure in SR piglets, in order to justify the use of the SR piglet as a model for the breast-fed human infant.

## Materials and Methods

### Animals, Housing, and Feeding

Thirty-nine naturally farrowed piglets (intact males, *n* = 20; females, *n* = 19) selected from 10 litters were obtained from the Imported Swine Research Laboratory located at the University of Illinois. Piglets were allowed access to colostrum for up to 48 h, at which point they were randomly allotted to one of two experimental treatments (AR intact males *n* = 15, AR females *n* = 14; SR intact males *n* = 5, SR females *n* = 5) for the duration of a 25-day feeding study (conducted using five cohorts of piglets). Average body weights were not different (*P* = 0.995) between treatments at allotment (AR piglets: 1.44 ± 0.218 kg; SR piglets: 1.43 ± 0.300 kg). AR piglets were housed individually in stainless steel cages and were provided with a towel and toy for comfort and environmental enrichment, and interacted with caretakers two to three times daily. Caging specifications have previously been described (15). Cages were outfitted with a heat lamp and electric heat mat (K&H Manufacturing, Colorado Springs, CO, USA) to maintain home cage temperatures between 23 and 31°C. Temperatures were gradually lowered throughout the study, as piglets were able to better regulate body temperature. Both SR and AR piglets were raised in the same facility and experienced similar temperature fluctuations. Additionally, a 12-h light/dark cycle was maintained with minimal light provided during dark cycles for the duration of the study for both SR and AR piglets.

Per agricultural protocols, all piglets were identified at birth using ear notches, and needle teeth were removed to prevent harm to littermates and the sow. Moreover, piglets received a supplemental iron injection at day 1 of age, but did not undergo other agricultural processing (i.e., tail docking and castrations). Approximately 5 ml of *Clostridium perfringens* antitoxin C + D (Colorado Serum Company, Denver, CO, USA) was administered subcutaneously as a prophylactic agent at day 2 of age. If piglets developed diarrhea, supplemental water and electrolytes (Pedialyte, Abbott Laboratories, Abbott Park, IL, USA) were provided. Sulfamethoxazole and trimethoprim oral suspension (50 and 8 mg/mL, respectively, Hi-Tech Pharmacal, Amityville, NY, USA) were given if symptoms were not alleviated after 48 h of exhibiting initial symptoms.

Artificially reared piglets were fed infant formula modified to meet or exceed the nutrient requirements of the piglet (18). Feedings were provided hourly using an automated feeding system over a period of 13 ± 2 h based on daily BW of piglets. The diets were replaced twice daily and were dispensed from six identical reservoirs that were cleaned and sterilized daily. Piglets were fed 285, 300, and 325 mL/kg BW from 0 to 7 days, 8 to 16 days, and 18 to 25 days on study, respectively. SR pigs remained with their respective mother and littermates for the duration of the study and only had access to maternal milk (i.e., no other sources of nutrition were accessible). All animal care and experimental procedures were in accordance with the Guide for the Care and Use of Laboratory Animals and approved by the Institutional Animal Care and Use Committee of the University of Illinois.

### Magnetic Resonance Imaging

#### Magnetic Resonance Imaging and Anesthesia Overview

All piglets underwent MRI procedures at 21 ± 2 days of age at the Beckman Institute Biomedical Imaging Center using a Siemens MAGNETOM Trio 3-T MRI, with a Siemens 12-channel head coil. Each piglet underwent imaging protocols only once, but scans for each cohort were completed over multiple days due to timing constraints. Piglets were randomly assigned a scan date and order to avoid bias. The piglet neuroimaging protocol included three magnetization prepared rapid gradient echo (MPRAGE) sequences and diffusion tensor imaging (DTI) to assess brain macrostructure and microstructure, respectively, as well as magnetic resonance spectroscopy (MRS) to obtain brain metabolite concentrations. In preparation for MRI procedures, anesthesia was induced using an intramuscular injection of telazol:ketamine:xylazine (50.0 mg of tiletamine plus 50.0 mg of zolazepam reconstituted with 2.50 mL ketamine (100 g/L) and 2.5 mL xylazine (100 g/L); Zoetis, Florham Park, NJ, USA) administered at 0.022 mL/kg BW, and maintained with inhalation of isoflurane (98% O_2_, 2% isoflurane). Piglets were immobilized during all MRI procedures. Visual observation of a subject’s well-being as well as observations of heart rate, PO_2_, and percent of isoflurane were recorded every 5 min during the procedure, and every 10 min post-procedure until animals recovered. Total scan time for each pig was approximately 60 min. Imaging techniques are briefly described below, while detailed methods for manual brain segmentation, volumetric assessment, voxel-based morphometry (VBM), and DTI were previously described (14–16).

#### Structural MRI Acquisition and Analysis

A T1-weighted MPRAGE sequence was used to obtain anatomic images of the piglet brain, with a 0.7 isotropic voxel size. Three repetitions were acquired and averaged using SPM8 in Matlab 8.3, and brains were manually extracted using FMRIB Software Library (FSL) (FMRIB Centre, Oxford, UK). The following sequence-specific parameters were used to acquire T1-weighted MPRAGE data: TR = 1900 ms; TE = 2.49 ms; 224 slices; FOV = 180 mm; flip angle = 9°. Methods for MPRAGE averaging, manual brain extraction were previously described (15). All data generated used a publicly available population-averaged piglet brain atlas (http://pigmri.illinois.edu) (19).

Voxel-based morphometry analysis was performed, to assess gray matter (GM) and WM tissue concentrations using SPM8 software (Wellcome Department of Clinical Neurology, London, UK). Manually extracted brains were aligned to piglet brain atlas space using a 12-parameter affine transformation. The “Segment” function of SPM and piglet-specific prior probability tissue maps were then used to segment the brains into GM and WM. The DARTEL toolbox was used with piglet-specific specifications that included changing the bounding box of −30.1 to 30.1, −35 to 44.8, −28 to 31.5, and a voxel size of 0.7 mm^3^. After the non-linear transformation of the data in the DARTEL procedure, flow fields were created and converted to warp files. The warp files generated were then applied to the subject’s GM and WM. The modulated data were smoothed with a 4-mm full-width half maximum (FWHM) and were subjected to VBM procedures using the statistical non-parametric methods toolbox (SnPM). For VBM analyses, two-sample permutation *t*-tests were performed on a voxel-by-voxel basis for GM and WM volume differences between all AR and SR animals with an uncorrected *P* < 0.001. An additional threshold criterion of at least 20-edge connected voxels was used.

For volumetric assessments, individual brains were segmented into 19 different regions of interest (ROIs) using the piglet brain atlas. Total brain and individual region volume analysis was performed in which an inverse warp file for each ROI was generated from the DARTEL-generated warp files for each region using the using the SPM software. Generation of region-specific warp files was previously described (14, 16). Due to differences in absolute whole brain volume, all ROIs were also expressed as a percent of total brain volume (%TBV), using the following equation: (region of interest absolute volume)/(total brain absolute volume) × 100, within subject.

#### Diffusion Tensor Imaging Acquisition and Analysis

Diffusion tensor imaging was used to assess WM maturation and axonal tract integrity using *b* value = 1000 s/mm^2^ across 30 directions and a 2 mm isotropic voxel. Diffusion-weighted EPI images were assessed in FSL for fractional anisotropy (FA), mean diffusivity (MD), axial diffusivity (AD), and radial diffusivity (RD) using methods previously described (15). Assessment was performed over the following ROIs: whole brain WM (generated from the Pig MRI atlas), DTI-generated WM, left and right cortices, corpus callosum, internal capsule, thalamus, and both hippocampi were performed using a customized piglet analysis pipeline and the FSL software package. For the purposes of this analysis, we used the Piglet Brain atlas, generated from the same species, developed by Conrad et al. (19). The diffusion toolbox in FSL was used to generate values of AD, RD, MD, and FA. In the corresponding results, atlas-generated WM indicates the use of WM prior probability maps from the piglet brain atlas, which were used as a ROI mask. Likewise, DTI-generated WM indicates a threshold of 0.2 was applied to FA values, thus restricting analysis to WM tracts.

Masks for each ROI from the atlas were non-linearly transformed into the MPRAGE space of each individual pig, and a linear transform was then applied to transfer each ROI into DTI space. A threshold of 0.5 was applied to each ROI, and the data were dilated twice. For each individual ROI, an FA threshold of 0.15 was applied to ensure that we included only WM in that ROI despite the mask expansion.

#### Magnetic Resonance Spectroscopy Acquisition and Analysis

**Figure 1:**
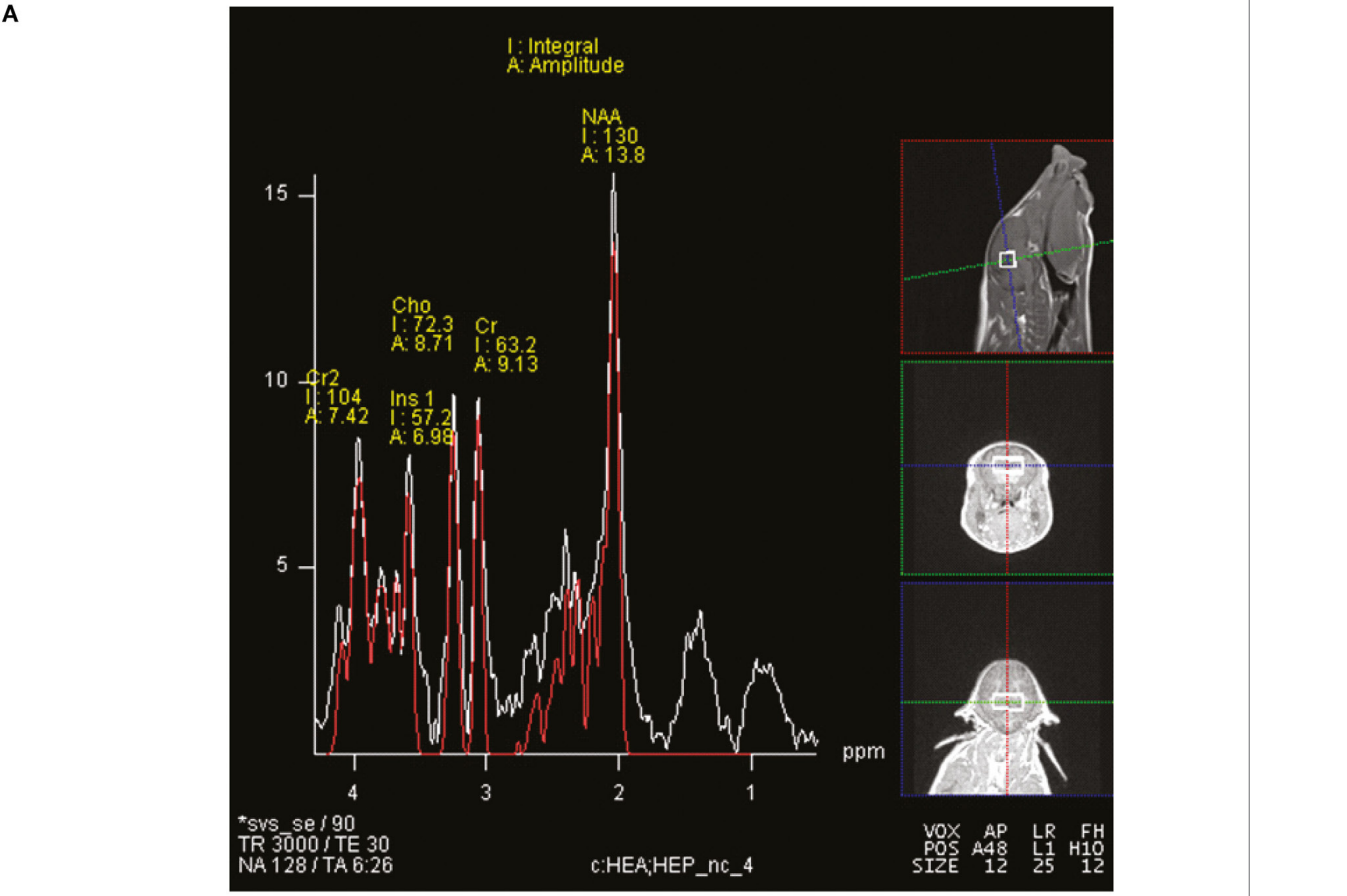

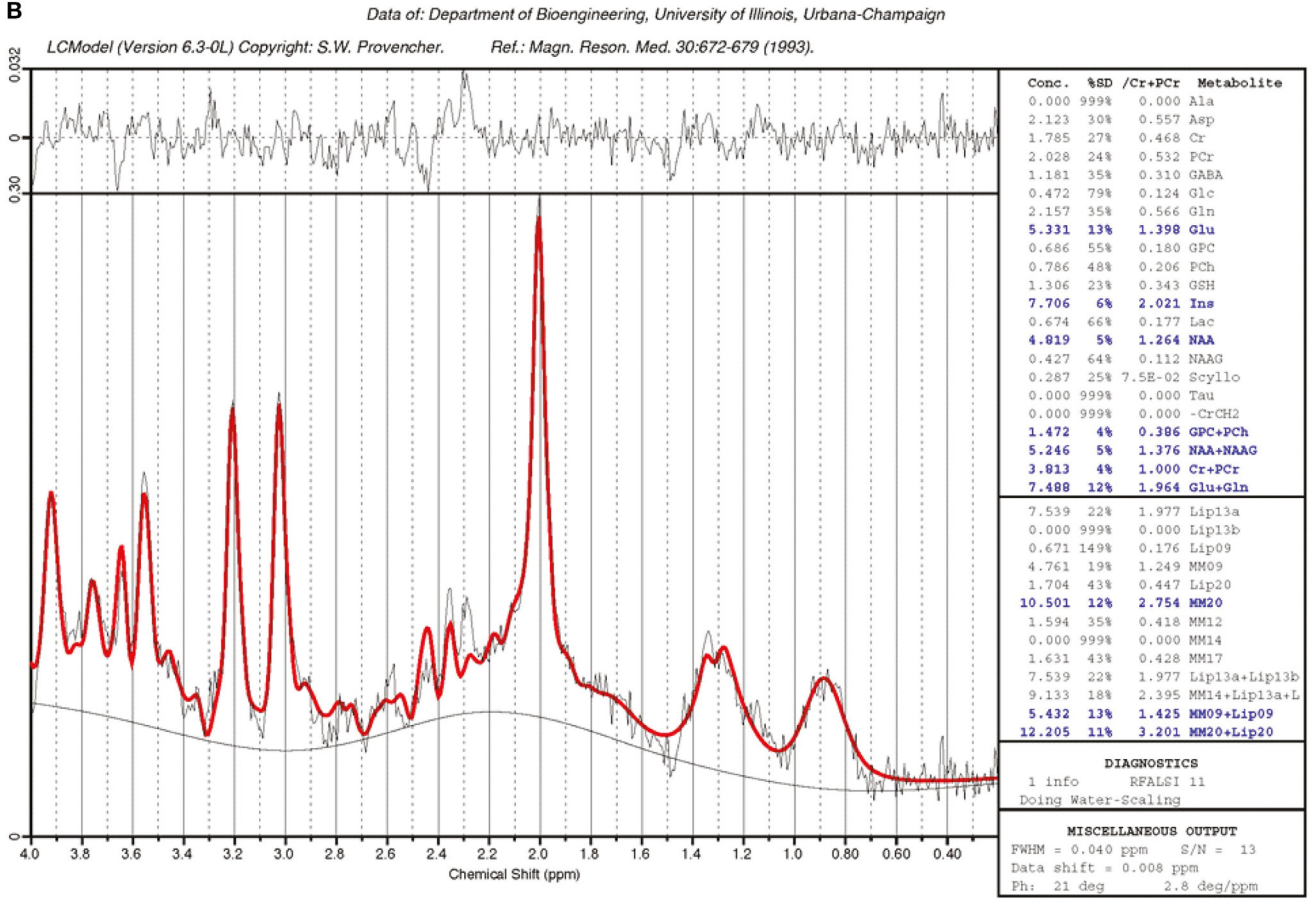
**Representative single-voxel spectroscopy spectrum**. Single-voxel spectroscopy was used to analyze brain metabolites in piglets. **(A)** The relative positioning of the voxel, which contains the hippocampi and interstitial space. **(B)** The acquired spectra with generated data to compare metabolites between AR and SR piglets. A threshold of Cramér–Rao < 20 was used for analyzed metabolites, data are presented as absolute concentrations in parts per million.

Magnetic resonance spectroscopy was used to non-invasively quantify metabolites both in hippocampi and in intervening tissue. The MRS spin-echo chemical shift sequence was used with a voxel size of 12 mm × 25 mm × 12 mm and centered over the left and right dorsal hippocampi (Figure 1). The following sequence parameters were used in acquisition of spectroscopy data: TR = 3000 ms; TE = 30 ms; signal averages = 128 (water-suppressed scan) and 8 (non-water-suppressed scan); vector size = 1024 point. Both water-suppressed and non-water-suppressed data were collected in institutional units, and all MRS data were analyzed using LC Model (version 6.3), using methods previously described (16). There were two limits placed on MRS data for inclusion in the statistical analysis. Cramér–Rao lower bounds (i.e., % SD) were calculated using the LC Model, and only metabolites with SD <20% were considered to have reliable quantitative results of absolute levels. In addition, metabolites included in the analysis were identified in at least four subjects per treatment. Metabolite concentrations were expressed in absolute and as a proportion of creatine values, but absolute concentrations were used for statistical analysis as creatine levels fluctuated between AR and SR piglets.

### Statistical Analysis

Overall, numerical data (i.e., brain volumes, DTI, and MRS) were subjected to an analysis of variance (ANOVA) with main effects of treatment and sex using the MIXED procedure in SAS v 9.4. Replicate cohort of pigs was included as a random variable, and the threshold of significance was set at *P* < 0.05. Data are presented as means ± SEM with significance accepted at *P* < 0.05.

## Results

### Piglet Growth

Piglet weights were not different between (*P* = 0.9951) dietary treatments upon allotment (AR piglets: 1.44 ± 0.218 kg; SR piglets: 1.43 ± 0.300 kg). Body weights were also recorded on the day each individual piglet was subjected to MRI procedures. Analysis of piglet weights on the day of MRI indicated differences due to diet (*P* < 0.001) (AR piglets: 3.79 ± 0.218 kg; SR piglets: 6.87 ± 0.300 kg). Over the course of the study, eight AR piglets received antibiotic treatment from days 9 to 11 of life, after which signs of diarrhea were alleviated.

### Estimated ROI Volumes

Estimation of absolute whole brain volumes revealed larger (*P* < 0.001) brains in SR piglets compared with AR piglets (Table 1). After normalizing ROIs to %TBV, SR piglets exhibited proportionally smaller (*P* < 0.05) volumes compared with AR piglets in the following regions: corpus callosum, caudate, hypothalamus, midbrain, olfactory bulb, pons, thalamus, left and right hippocampi, and left and right cortices. Ventricular system components, including the cerebral aqueduct, fourth ventricle, lateral ventricle, and third ventricle, were also smaller (*P* < 0.05) as a %TBV in SR piglets when compared with AR piglets. A main effect of sex (*P* < 0.05) in which female piglets exhibited larger regions as a %TBV compared with male piglets was observed in the following regions: GM, WM, corpus callosum, caudate, hypothalamus, internal capsule, left/right cortices, olfactory bulb, putamen, and thalamus.

**Table 1:** **Region-specific absolute and relative volumes for artificially reared (AR) and sow-reared (SR) piglets**.^a^

	Absolute (mm^3^)		*P*-value		Relative (%TBV)^b^		*P*-value	
Brain region	AR	SR	Treatment	Sex	AR	SR	Treatment	Sex
Whole brain	58730 ± 3046	73199 ± 3458	<0.001	0.163	–	–	–	–
Gray matter	29677 ± 786	34585 ± 939	<0.001	0.202	50.87 ± 1.412	48.25 ± 1.831	0.128	0.021
White matter	11155 ± 517	13999 ± 586	<0.001	0.108	19.07 ± 0.518	19.43 ± 0.719	0.619	0.008
Cerebral aqueduct	117 ± 2.0	118 ± 3.3	0.643	0.351	0.20 ± 0.009	0.17 ± 0.011	<0.001	0.622
Cerebellum	4581 ± 138	5372 ± 154	<0.001	0.821	7.85 ± 0.185	7.47 ± 0.248	0.120	0.081
Corpus callosum	1032 ± 29.5	1167 ± 34.5	<0.001	0.198	1.77 ± 0.054	1.62 ± 0.066	0.010	0.016
Caudate	522 ± 15.3	552 ± 17.7	0.025	0.187	0.90 ± 0.034	0.77 ± 0.039	<0.001	0.031
Fourth ventricle	138 ± 2.69	151 ± 3.66	<0.001	0.611	0.24 ± 0.010	0.21 ± 0.012	0.01	0.397
Hypothalamus	529 ± 14.1	602 ± 16.0	<0.001	0.377	0.91 ± 0.033	0.84 ± 0.038	0.015	0.04
Internal capsule	4734 ± 168	5641 ± 189	<0.001	0.131	8.12 ± 0.235	7.85 ± 0.305	0.342	0.018
Left cortex	16364 ± 385	18504 ± 437	<0.001	0.216	28.07 ± 0.769	25.80 ± 0.942	0.007	0.019
Left hippocampus	574 ± 12.9	627 ± 15.4	<0.001	0.906	0.99 ± 0.032	0.87 ± 0.039	0.001	0.185
Lateral ventricle	1117 ± 21.5	1110 ± 33.1	0.854	0.868	1.93 ± 0.098	1.55 ± 0.112	<0.001	0.42
Midbrain	2975 ± 71.5	3318 ± 80.6	<0.001	0.532	5.11 ± 0.151	4.62 ± 0.181	0.002	0.055
Medulla	2377 ± 62.5	2744 ± 70.4	<0.001	0.399	4.08 ± 0.108	3.83 ± 0.141	0.064	0.054
Olfactory bulb	2948 ± 59.1	3332 ± 71.7	<0.001	0.261	5.06 ± 0.131	4.65 ± 0.167	0.010	0.024
Pons	849 ± 16.7	918 ± 18.4	<0.001	0.454	1.46 ± 0.047	1.29 ± 0.057	0.001	0.093
Putamen	1130 ± 31.0	1301 ± 36.2	<0.001	0.086	1.94 ± 0.062	1.81 ± 0.077	0.061	0.016
Right cortex	15479 ± 346	17588 ± 394	<0.001	0.204	26.56 ± 0.747	24.53 ± 0.911	0.011	0.021
Right hippocampus	631 ± 16.2	700 ± 19.3	<0.001	0.819	1.08 ± 0.036	0.97 ± 0.042	0.002	0.098
Thalamus	2960 ± 65.1	3340 ± 76.4	<0.001	0.176	5.09 ± 0.162	4.66 ± 0.198	0.013	0.035
Third ventricle	207 ± 4.21	216 ± 5.56	0.104	0.829	0.36 ± 0.014	0.30 ± 0.016	<0.001	0.197

*^a^Values are presented as treatment mean ± SEM. Experimental treatments include artificially reared (AR; male *n* = 15; female *n* = 14) and sow-reared (SR; male *n* = 5; female *n* = 5) piglets*.

*^b^Percent total brain volume (%TBV) was calculated as following: (brain region absolute volume/whole brain absolute volume) × 100*.

### Voxel-Based Morphometry

**Figure 2:**
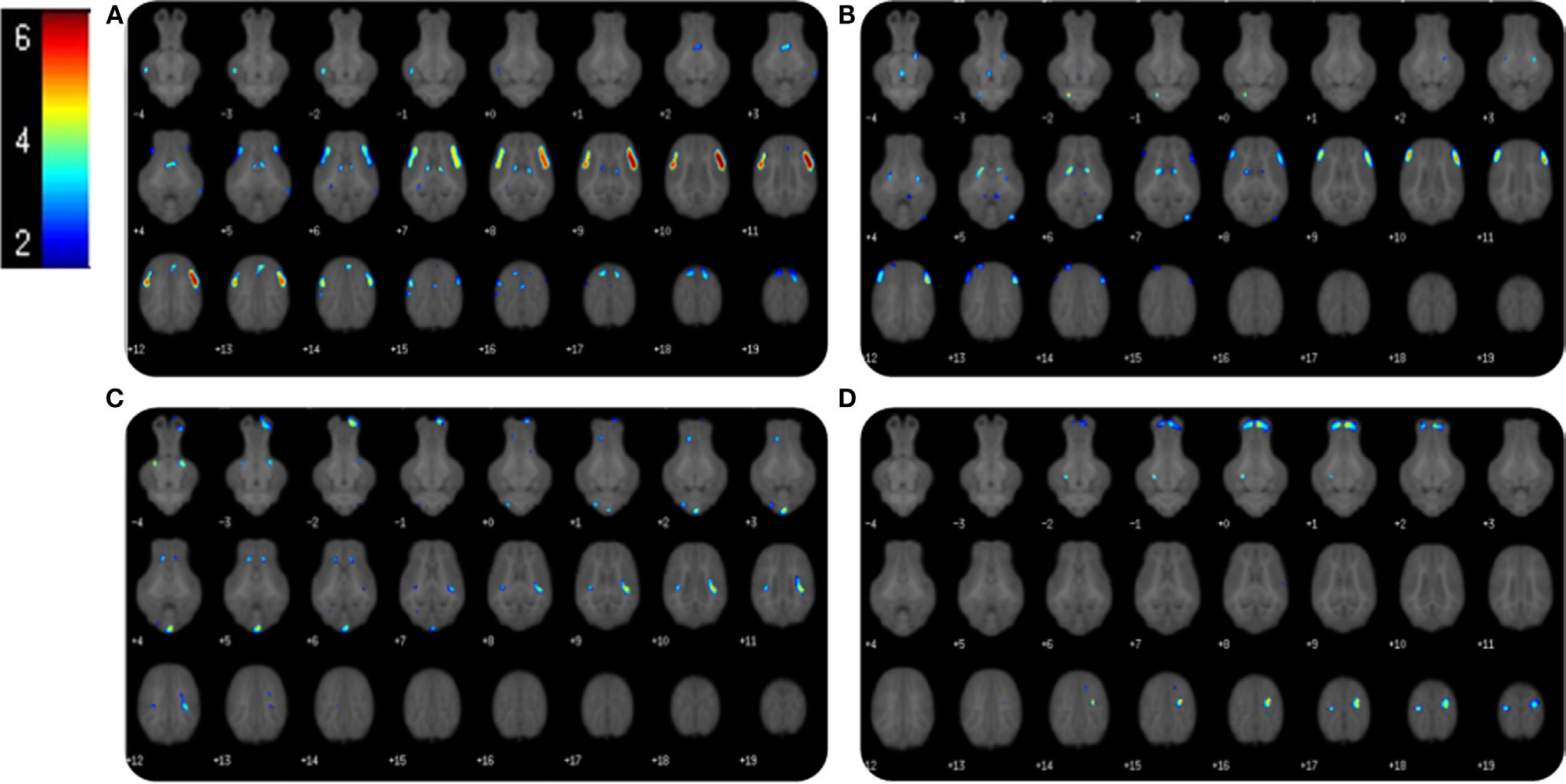
**Voxel-based morphometry of AR and SR piglets**. Voxel-based morphometric analysis of white and gray matter volume differences between the average of artificially reared (AR) and sow-reared (SR) piglets. Clusters <20 voxels, or voxel clusters found on the edge of the brain, were excluded from the analysis, and height threshold was set at *P* = 0.001. **(A)** Voxel clusters in which AR > SR in gray matter volume; **(B)** voxel clusters in which AR > SR in white matters volume; **(C)** voxel clusters in which SR > AR in gray matter volume; **(D)** voxel clusters in which SR > AR in white matter volume. Heat map indicated pseudo-*t* value from 2 to 6. Slice identifiers based off of Pig Brain Atlas, available at http://pigmri.illinois.edu/, Conrad et al. (19).

Voxel-based morphometry analysis of GM concentrations revealed higher regional peak intensities (*P* < 0.05) in AR piglets when compared with SR piglets (Figure 2; Table 2). Within these significant comparisons, AR piglets exhibited significantly larger GM voxel clusters predominantly in the right and left cortices. However, it should be noted that significant voxel clusters where SR had larger (*P* < 0.05) GM concentration compared with AR piglets were also observed, although to a lesser extent and with smaller voxel clusters. Analysis of WM concentrations also revealed differences (*P* < 0.05) due to diet, in which AR piglets exhibited higher WM regional peak intensities compared with SR piglets. When compared with SR piglets, AR piglets exhibited significantly larger WM voxel clusters predominantly in the left and right cortices (Figure 2; Table 3). Again, it should be noted that significant voxel clusters where SR had higher (*P* < 0.05) WM concentrations compared with AR piglets were also observed.

**Table 2:** **Comparison of gray matter cluster volumes between artificially reared (AR) and sow-reared (SR) piglets**.^a^

Comparison	Anatomic region	Cluster (voxels)	Peak-level (*P*-value)	*x*	*y*	*z*	Pseudo-*t*
SR > AR	Right cortex	657	0.0002	14	3	10	5.44
	Right cortex^b^		0.0008	10	12	13	3.07
	Olfactory	417	0.0004	−4	25	−8	5.29
	Undefined	85	0.0002	−9	8	−4	5.26
	Right cortex	356	0.0004	10	7	−6	5.26
	Cerebellum	398	0.0002	2	−23	4	5.2
	Undefined	1032	0.0008	6	34	−1	5.06
	Cerebellum	149	0.0008	−7	−20	0	4.18
	Left cortex	203	0.0002	−4	24	3	3.96
	Left cortex	164	0.0004	−12	4	10	3.88
	Undefined	74	0.0006	5	24	4	3.69
	Medulla	60	0.0008	3	−22	−15	3.62
	Cerebellum	24	0.0008	9	−20	−2	3.03
AR > SR	Right cortex	2149	0.0002	17	14	10	7.98
	Left cortex	1619	0.0002	−16	14	10	6.54
	Left cortex^b^		0.0004	−13	24	8	5.33
	Caudate	469	0.0008	5	10	7	4.82
	Left cortex	272	0.0002	−17	−1	−4	4.49
	Right cortex	148	0.0004	3	24	13	4.29
	Right cortex	463	0.0008	6	17	17	4.04
	Left cortex	315	0.0002	−4	19	17	4.01
	Left cortex	97	0.0008	−17	6	14	3.51
	Left cortex	56	0.0002	1	10	15	3.36
	Right cortex	90	0.0002	21	−5	5	3.02
	Undefined	53	0.0004	−10	−2	8	2.88
	Right cortex	23	0.0008	20	5	13	2.74

*Clusters > 20 were included in analysis with threshold set at P < 0.001, uncorrected P-values presented*.

*^a^Experimental treatments include AR (*n* = 29) and SR (*n* = 10) groups*.

*^b^Missing cluster value is contiguous with voxel cluster listed immediately above and represents 1 of 2 continuous voxel clusters with 2 different center voxels*.

**Table 3:** **Comparison of white matter cluster volumes between artificially reared (AR) and sow-reared (SR) piglets**.^a^

Comparison	Anatomic region	Cluster (voxels)	Peak-level (*P*-value)	*x*	*y*	*z*	Pseudo-*t*
SR > AR	Right cortex	522	0.0008	10	10	15	5.66
	Right cortex	1211	0.0002	3	34	1	5.45
	Left cortex	191	0.0006	−10	6	17	4.93
	Thalamus	94	0.0004	−8	−1	−1	4.68
	Olfactory	272	0.0006	4	23	−8	4.34
	Right cortex	22	0.0008	6	20	15	3.27
	Undefined	24	0.0006	1	−27	−8	0.09
AR > SR	Right cortex	1553	0.0002	17	20	10	6.05
	Left cortex	1863	0.0002	−15	24	10	5.9
	Left cortex^b^		0.0002	−7	28	13	3.23
	Cerebellum	80	0.0006	−7	−15	−1	5.37
	Internal capsule	117	0.0002	7	13	6	5.17
	Undefined	285	0.0008	−6	15	5	5
	Right cortex	348	0.0006	10	9	−5	4.96
	Right cortex	514	0.0002	14	−18	6	4.39
	Midbrain	224	0.0002	0	−2	−5	4.11
	Internal capsule	77	0.0004	10	8	3	4.1
	Midbrain	79	0.0008	5	−5	3	2.68
	Right cortex	483	0.0008	13	30	9	2.62
	Medulla	252	0.0008	2	−12	−17	2.61
	Undefined	28	0.0006	−4	−4	4	2.41
	Cerebellum	31	0.0008	−4	−18	4	2.26
	Right cortex	50	0.0008	6	16	19	1.42

*Clusters > 20 were included in analysis with threshold set at P < 0.001, uncorrected P-values presented*.

*^a^Experimental treatments include AR (*n* = 29) and SR (*n* = 10) groups*.

*^b^Missing cluster value is contiguous with voxel cluster listed immediately above and represents 1 of 2 continuous voxel clusters with 2 different center voxels*.

### Diffusion Tensor Imaging

Due to excessive motion, two piglets (one AR male and one SR female) were not included in this analysis. Diffusion tensor analysis revealed higher (*P* < 0.05) FA values in SR piglets compared with AR piglets in the following regions: atlas-generated WM, DTI-generated WM, internal capsule, left cortex, and right cortex (Table 4). Observed MD measures in SR piglets were lower (*P* < 0.05) in the atlas-generated WM and right cortex compared with AR piglets. Analysis of RD revealed decreased (*P* < 0.05) rates of diffusion in SR piglets compared with AR piglets in the following regions: atlas-generated WM, DTI-generated WM, internal capsule, and right cortex. AD of atlas-generated WM was lower (*P* < 0.05) in SR piglets compared with AR piglets, and higher (*P* < 0.05) in the internal capsule in SR piglets compared with AR piglets. A main effect of sex was detected in the right hippocampus, with female piglets exhibiting higher (*P* < 0.05) AD and MD values when compared with male piglets.

**Table 4:** **Diffusion tensor imaging (DTI) values for artificially reared (AR) and sow-reared (SR) piglets**.^a^

	Experimental treatment		*P*-value	
	AR	SR	Treatment	Sex
**Fractional anisotropy^b^**				
White matter from atlas^c^	0.3000 ± 0.0011	0.3084 ± 0.0019	0.0005	0.6173
White matter from DTI^d^	0.2989 ± 0.0011	0.3091 ± 0.0019	<0.0001	0.4217
Corpus callosum	0.2766 ± 0.0034	0.2851 ± 0.0060	0.2253	0.3099
Internal capsule	0.3839 ± 0.0044	0.4123 ± 0.0076	0.0029	0.1462
Left cortex	0.2957 ± 0.0028	0.3106 ± 0.0050	0.0148	0.6764
Left hippocampus	0.2701 ± 0.0049	0.2784 ± 0.0072	0.2754	0.1063
Right cortex	0.2979 ± 0.0014	0.3084 ± 0.0023	0.0005	0.9841
Right hippocampus	0.2719 ± 0.0034	0.2734 ± 0.0060	0.8233	0.0862
Thalamus	0.3099 ± 0.0035	0.3204 ± 0.0053	0.0683	0.8425
**Mean diffusivity^b^**				
White matter from atlas	1.046 ± 0.019	1.007 ± 0.020	0.0005	0.3526
White matter from DTI	1.008 ± 0.018	0.985 ± 0.02	0.0565	0.7453
Corpus callosum	1.247 ± 0.021	1.205 ± 0.038	0.3345	0.8696
Internal capsule	0.871 ± 0.004	0.870 ± 0.006	0.9798	0.4924
Left cortex	1.030 ± 0.018	0.988 ± 0.030	0.2231	0.3477
Left hippocampus	1.110 ± 0.046	1.088 ± 0.054	0.5886	0.5867
Right cortex	1.000 ± 0.015	0.980 ± 0.016	0.0497	0.9418
Right hippocampus	1.048 ± 0.024	1.082 ± 0.043	0.4883	0.0563
Thalamus	0.911 ± 0.011	0.919 ± 0.016	0.6433	0.1602
**Axial diffusivity^b^**				
White matter from atlas	1.380 ± 0.022	1.339 ± 0.024	0.0005	0.2741
White matter from DTI	1.330 ± 0.021	1.313 ± 0.023	0.1901	0.8264
Corpus callosum	1.613 ± 0.031	1.566 ± 0.054	0.4594	0.8707
Internal capsule	1.250 ± 0.006	1.287 ± 0.011	0.0062	0.5208
Left cortex	1.354 ± 0.022	1.321 ± 0.034	0.3633	0.3737
Left hippocampus	1.435 ± 0.057	1.423 ± 0.068	0.8086	0.9655
Right cortex	1.318 ± 0.018	1.306 ± 0.019	0.2933	0.9194
Right hippocampus	1.361 ± 0.033	1.397 ± 0.058	0.5869	0.0326
Thalamus	1.222 ± 0.018	1.246 ± 0.026	0.3428	0.1686
**Radial diffusivity^b^**				
White matter from atlas	0.879 ± 0.017	0.841 ± 0.019	0.0005	0.4111
White matter from DTI	0.847 ± 0.016	0.821 ± 0.018	0.0272	0.7058
Corpus callosum	1.064 ± 0.017	1.024 ± 0.030	0.2512	0.6493
Internal capsule	0.681 ± 0.005	0.662 ± 0.007	0.0226	0.1455
Left cortex	0.868 ± 0.017	0.822 ± 0.029	0.1678	0.3403
Left hippocampus	0.947 ± 0.041	0.921 ± 0.047	0.4526	0.3399
Right cortex	0.840 ± 0.014	0.817 ± 0.015	0.0159	0.9480
Right hippocampus	0.891 ± 0.021	0.926 ± 0.037	0.4098	0.1050
Thalamus	0.756 ± 0.007	0.755 ± 0.012	0.9731	0.1922

*^a^Values are presented as means ± SEM. Experimental treatments include artificially reared (AR; male *n* = 14; female *n* = 14) and sow-reared (SR; male *n* = 5; female *n* = 4) groups*.

*^b^Absolute values for fractional anisotropy are presented arbitrary units, while mean diffusivity, axial diffusivity, and radial diffusivity are presented as ×1000 mm^2^/s*.

*^c^White matter generated from atlas indicates the use of white matter prior probability maps generated from the Piglet Brain atlas were used as an region of interest mask*.

*^d^White matter generated from DTI indicates a threshold of 0.2 was applied to FA values, thus restricting analysis to white matter tracts*.

### Magnetic Resonance Spectroscopy

Due to excessive motion, four piglets (two AR male and two SR female) were not included in this analysis. MRS analysis resulted in quantification of eight brain metabolites (Table 5). Of the observed metabolites, SR piglets exhibited higher (*P* < 0.05) concentrations of creatine + phosphocreatine, glycerophosphocholine + phosphocholine, and myo-inositol compared with AR piglets. However, AR piglets exhibited higher (*P* < 0.05) concentrations of glutamate + glutamine compared with SR piglets.

**Table 5:** **Hippocampal metabolite concentrations for artificially reared (AR) and sow-reared (SR) piglets**.^a^

	Experimental treatment		*P*-value	
Metabolite	AR	SR	Treatment	Sex
Creatine + Phosphocreatine	3.48 ± 0.090 (26)	4.22 ± 0.162 (8)	<0.001	0.830
Glutamate	5.70 ± 0.364 (26)	5.30 ± 0.592 (5)	0.484	0.005
Glutamate + Glutamine	9.43 ± 0.480 (27)	7.43 ± 0.772 (8)	0.021	0.020
Glycerophosphocholine + Phosphocholine	1.28 ± 0.045 (27)	1.65 ± 0.083 (8)	0.001	0.430
Glutathione	2.34 ± 0.196 (16)	2.29 ± 0.365 (5)	0.897	0.646
Myo-inositol	8.04 ± 0.240 (27)	9.43 ± 0.409 (8)	0.004	0.504
*N*-acetylaspartate	4.80 ± 0.185 (26)	4.98 ± 0.286 (8)	0.548	0.562
*N*-acetylaspartate + *N*-acetylaspartylglutamate	5.27 ± 0.131 (26)	5.66 ± 0.232 (8)	0.150	0.458

*^a^Values are presented as means ± SEM. Single-voxel MRS for metabolites met criteria for analysis (<20% SD) and were detected in at least four pigs per treatment. Experimental treatments include artificially reared (AR; male *n* = 13; female *n* = 14) and sow-reared (SR; male *n* = 5; female *n* = 3) groups; sample size is denoted in parentheses*.

## Discussion

This study employed MRI techniques to assess the impact of early life nutrition on macro- and microstructural development of the neonatal piglet brain. AR animals were raised on infant formula, altered to meet the nutrient requirements of the piglet, while SR animals received sow’s milk for the duration for the study. Quantification of neurodevelopmental differences between SR and AR piglets were characterized by employing three different MRI techniques. Analyses revealed larger whole brain volumes and proportionally smaller ROIs in SR piglets, differences in diffusion tensor measures between treatments, and altered concentrations of metabolites indicative of accelerated neurodevelopmental trajectory in SR piglets at approximately 3 weeks of age.

The authors acknowledge that the relative social isolation of AR piglets compared with their SR counterparts may be a confounding factor in this study. Group rearing was ruled out in favor of single housing, as metabolic outcomes are commonly required for studies involving assessment of novel compounds added to formula diets. To alleviate the potential impact of social isolation, AR piglets were given opportunities to engage in play with caretakers two to three times per day for the duration of the study. Furthermore, the AR piglet has previously been established as a normative model in nutrition studies (20). It is expected that the piglet will continue to be used in the field of nutrition and nutritional neuroscience heavily. As such, our study design was purposely engineered to establish baseline data in AR piglets compared with SR piglets.

### Volumetric Assessment

Over the course of the study, piglet growth differed by dietary treatment. While AR piglets were smaller compared with SR piglets at time of MRI assessment, AR piglet weights tend to vary between studies and these weights are within the typical range observed for AR piglets (16). Assessment of absolute brain volumes suggested larger brains in SR piglets compared with AR piglets, which may be attributable to differences in overall body growth of the piglets. Due to the difference in total absolute brain volumes, we chose to express ROI volumes relative to whole brain volume in an effort to obtain a more sensitive assessment of regional differences, which is consistent with human neuroimaging procedures. As a %TBV, AR piglets exhibited 11 distinct brain regions and 4 ventricular volumes that were different compared with SR piglets. ROIs, including the left and right hippocampi, left and right cortices, caudate, midbrain, pons, putamen, thalamus, cerebral aqueduct, as well as the fourth, lateral, and third ventricles, were all proportionally smaller in SR animals when compared with AR animals. When assessing relative brain volume changes, the majority of regions analyzed were subcortical and proportionally larger in AR piglets. By expressing the volume of each discrete brain region as a proportion of total brain volume for each piglet, this approach accounts for differences in total brain volume between AR and SR piglets. Thus, the observed increase in relative proportion of subcortical regions must have been mirrored by a decrease in the relative proportion of other tissues. While we speculate this proportional decrease would have occurred in cortical regions, no differences existed between AR and SR piglets for regions considered cortical in nature. To address the lack of differences in relative cortical volumes, we provide the following limitations to our analysis.

While use of the piglet brain atlas allows for assessment of specific regions within the brain, the authors acknowledge that it is not as sensitive as human and rodent brain atlases. The piglet brain atlas is conservative in the brain regions that are parcellated, in which only easily defined brain regions were included. This inherently leaves interstitial tissue to not be included because it cannot be definitively attributed to specific brain regions. The observed relative values of brain regions in the SR piglets are consistently lower compared with AR piglets. Considering that SR piglets had larger whole brains, we propose that growth and expansion within unsampled space might be driving these decreases in relative values. Future research should focus on more extensive characterization of piglet brain regions to update the brain atlas and include more of the unaccounted interstitial space. Another limitation in our current VBM analysis protocol remains, as it does not allow for separation of cerebrospinal fluid (CSF) as a whole. Although ROI volumes for lateral, third, and fourth ventricle spaces were assessed and found to be higher in the AR group, a holistic picture of CSF-containing space is necessary to accurately interpret this result.

Differences due to sex were also observed in 11 of the 19 ROI assessed, characterized by larger relative volumes in female piglets when compared with males. Previous research using the piglet model revealed overall larger whole brain and regional volumes in males at sexual maturity, yet females reached maximum growth rate of brain regions at an earlier timepoint in the neurodevelopmental timeline (21). These volumetric observations are similar to differences observed in human infants in which males tend to exhibit larger absolute brain regions compared with females (22). Our assessment analyzed differences between sexes in a larger number of ROI than prior piglet studies. As such, our data may offer a starting point for further longitudinal assessment of the structural and functional differences that maybe present between males and females during infancy.

### Voxel-Based Morphometry

Voxel-based morphometry analysis revealed volumetric differences in both absolute GM and WM volume concentrations between AR and SR groups. Overall, we observed highly significant differences as indicated by increases in GM volume in the frontal–cortical regions of AR animals. Most significant GM clusters observed in AR piglets were located bilaterally in the rostral part of the brain. Larger voxel clusters of WM were also evident in the left and right cortices of AR animals. Since neurons are largely established prenatally, followed by extensive growth, and expansion in the postnatal period, it is possible that the observed differences in tissue concentrations are due to altered rates of cortical expansion which is occurring at this stage in development (9, 21). Additionally, as the brain is developing, tissue is reorganized, and axons are pruned away (23, 24). Given that the SR piglets exhibited larger total brains, it is possible that the cortical GM and WM concentration differences were due to this phenomenon of expansion and pruning of neurons. In contrast, AR piglets appeared to have a higher density of GM and WM in the cortices and exhibited smaller total brain volumes. Thus, it is possible that AR piglets had yet to experience the expansion as proposed for the SR piglets. These findings serve to corroborate the proposed volumetric findings presented above, both of which suggest enhanced growth and expansion in the SR piglets when compared with the AR piglets. To the best of our knowledge, no infant research has utilized VBM to characterize GM and WM concentration changes early in life, therefore not allowing for direct comparison between the young pig and human. Further research is needed to investigate the exact timing of cortical expansion and axon pruning in the piglet model.

### Diffusion Tensor Imaging

Diffusion parameters were used to characterize the organization and structural integrity of axonal tracts in the piglet brain. Of these parameters, FA serves as an orientation independent means of assessing anistotropic diffusion of water molecules; providing an indirect, yet sensitive, means of assessing WM development (25). MD values presented here account the averaged apparent diffusion coefficient within axonal tracts and represent a holistic picture of water diffusivity (26). Additionally, AD and RD diffusion rates represent diffusion along and across a fiber orientation, respectively, as components of MD. Because the DTI metrics are interdependent with AD and RD representing the diffusion along the principal direction of diffusion and orthogonally and FA representing the fraction of diffusion along the principal direction, we are able to speculate on tissue changes from these measures.

Higher FA values of the atlas-generated WM, DTI-generated WM, internal capsule, and left and right cortices were observed in SR piglets when compared with AR piglets. While these results may be indicative of myelination, additional elements such as clustering of Na^+^ channels, quantification of pre-oligodendrocyte to immature oligodendrocyte ratio, and electrophysiological properties should be assessed before attributing these results solely to myelination (25). Further exploration of underlying brain microstructure must be completed before differentiating between anisotropic diffusion that occurs due to myelination and increasing complexity in the organization of neuronal fibers. A reduction in whole brain and region-specific RD in WM has been previously linked to packing of fibers that occur before myelination (27). Similarly, higher AD and lower MD values of whole brain and specific ROI may be due to greater level of WM tract organization, rather than myelination alone.

In human infant studies, high FA and low MD values are observed in early-maturing fiber bundles, such as the internal capsule (28). An increase in FA, partnered with a reduction in MD, has been shown to indicate an increase in axonal density, pre-myelination, and myelination of established axonal tracts (29). Of the ROIs assessed in our study, FA values of the internal capsule were highest in both treatment groups, indicating a similar pattern of WM tract development in the piglet when compared with the human infant. Overall, our DTI assessments suggest that SR piglets may have more structural complexity of neuronal fiber organization and possibly myelination. However, more detailed tissue analysis is warranted to decipher whether myelination or density of fiber tracts contribute more to the anisotropic diffusion patterns observed.

Furthermore, higher AD values present in the internal capsule of SR animals may indicate greater axonal connectivity, while lower AD values of the atlas-generated WM might indicate the effects of pruning. Pruning or competitive elimination refers to environmentally regulated changes in the density of synapses per unit of dendritic length, as well as axonal pruning (30). Pruning seems to facilitate higher order functioning in humans, and typical patterns of neurodevelopment dictates that large scale synaptogenesis, followed by pruning of non-established connections, occurs before large scale myelination (23). These data further corroborate VBM findings, suggesting that SR piglets may have experienced more axonal pruning around the time of imaging compared with AR piglets. Accordingly, the reduction in GM concentrations (assessed through VBM) and atlas-generated WM AD values may suggest a reduction in axonal connections, indicating that SR animals might have experienced a greater degree of pruning when compared with AR animals. Higher AD values were also observed in the right hippocampus of female piglets when compared with males. When partnered with longitudinal data suggesting an accelerated developmental trajectory in female animals, as well as trending ROI data indicating larger hippocampal volumes in female animals, these data suggest greater axonal maturity in the right hippocampus of female piglets (21). Moreover, the elevated FA values in partnership with a reduction in atlas-generated RD suggest that the tracts of the SR animals may be more myelinated and are further along in neurodevelopment at approximately 3 weeks of age. While our data are consistent with human infant studies suggesting altered rates of WM development in breast-fed and formula-fed infants, our interpretation of these data remains speculative, as we did not directly assess pruning in our study. Future research using histological staining to correlate stages of myelination with DTI measures is needed to corroborate this hypothesis.

### Single-Voxel Spectroscopy

Magnetic resonance spectroscopy provides a measurement of brain metabolites that serve as biomarkers for metabolic efficiency, energy storage, inflammation, structural integrity, and brain integrity (31). Our data revealed total creatine (i.e., creatine + phosphocreatine) concentrations to be higher in SR piglets when compared with AR piglets. A primary role for the creatine system is to store and distribute phosphate-bound energy, thereby serving as a buffer system during low ATP:ADP status (32). During a low-energy state, phosphocreatine is paired with ADP, and a phosphate is transferred to ADP to generate creatine and ATP. In high-energy states, the reverse reaction occurs. Higher levels of total creatine may indicate higher energy stores in the SR piglets when compared with AR piglets.

Choline metabolites were also analyzed using MRS. Choline is important in neurodevelopment, as a precursor for the neurotransmitter acetylcholine, in cell membrane synthesis as a precursor of phosphatidylcholine, and is stored in cellular cytoplasm as phosphocholine. Our data suggest that glycerophosphocholine–phosphocholine levels were higher in SR animals, and this bound form of choline serves as choline storage molecules within the cell, which can then be used as a precursor in myelin synthesis (32). Thus, increased choline concentrations in SR animals may suggest pre-myelination events, which may corroborate the need for higher energy stores (i.e., increased total creatine) to support this metabolically taxing event. Spectroscopy findings also revealed higher concentrations of myo-inositol in SR piglets when compared with AR piglets. As myo-inositol serves as a precursor to the glycerophospholipid phosphatidylinositol, elevated levels of myo-inositol may suggest higher glial cell density in SR animals (31).

Additionally, quantification of glutamate–glutamine (GLU–GLN) is indicative of the abundance of the excitatory neurotransmitter GLU, and the degree of glucose consumption in the brain (33). In fact, the majority of glucose consumption in the brain occurs due to glutamatergic neural activity (33). A large proportion of glutamate in the brain is localized to neurons and is considered a slow-turnover pool, while smaller quantities of glutamate exist in glial cells. Extracellular glutamate is highly regulated and is preferentially shuttled into astrocytes, resynthesized into glutamine, and reused (31). In WM, an elevation in extracellular glutamate may be signaling proliferation and migration of preoligodendrocytes prior to differentiation into myelin producing entities (34, 35). Our data show greater GLU–GLN concentrations in AR piglets compared with SR piglets. Provided the limitations of not knowing whether or not this concentration is primarily intra- or extracellular, it is unclear what the significance of this finding might be in the context of the neurodevelopmental process.

## Conclusion

To the best of our knowledge, this study is the first of its kind to characterize neurodevelopmental patterns of the SR piglet. Although only a single timepoint in the invariably changing neonatal neural network was studied, these data provide a foundation for establishing the SR piglet as a normative standard when using the AR piglet as a biomedical species for studying nutritional neuroscience. The authors note that differences in rearing environment and weight of piglets may have also had an impact on observed brain development outcomes. However, this style of rearing and the difference in weight between AR and SR are commonly observed in nutritional neuroscience studies using the piglet model. Thus, it is important to establish a baseline characterization in the brain development of these animals as their use in this field is of relevance. Notable evidence from our DTI measures indicates similarities between SR piglet brain development and breast-fed infant brain development, further justifying this animal model for translational research. Overall results of this study suggest SR piglets exhibit larger whole brain volumes and greater WM maturation at approximately 3 weeks of age compared with their AR counterparts. However, behavioral assessments are required to ascertain functional implications of these variations in brain macro- and microstructure. It is important to note that these data could lay a foundation for future nutritional neuroscience research using the piglet.

## Author Contributions

RD and C-SL were involved in project conceptualization. RJ and LA were involved in daily project activities. RJ, AM, LA, and RD were involved in data collection and data analysis. All authors were involved in data interpretation and manuscript preparation.

## Conflict of Interest Statement

RD received grant funding from Abbott Nutrition. C-SL is an employee of Abbott Nutrition. The remaining authors declare that the research was conducted in the absence of any commercial or financial relationships that could be construed as a potential conflict of interest.

## Abbreviations

% TBV, percent total brain volume; AD, axial diffusivity; AR, artificially reared; CR-PCR, creatine + phosphocreatine; DTI, diffusion tensor imaging; FA, fractional anisotropy; GLU–GLN, glutamate–glutamine; GM, gray matter; MD, mean diffusivity; MI, myo-inositol; MPRAGE, magnetization prepared rapid gradient echo; MRI, magnetic resonance imaging; MRS, magnetic resonance spectroscopy; RD, radial diffusivity; ROI, region of interest; SR, sow-reared; WM, white matter.
